# Long-Term Behaviour of Fly Ash and Slag Cement Grouts for Micropiles Exposed to a Sulphate Aggressive Medium

**DOI:** 10.3390/ma10060598

**Published:** 2017-05-30

**Authors:** José Marcos Ortega, María Dolores Esteban, Raúl Rubén Rodríguez, José Luis Pastor, Francisco José Ibanco, Isidro Sánchez, Miguel Ángel Climent

**Affiliations:** 1 Departamento de Ingeniería Civil, Universidad de Alicante, Ap. Correos 99, 03080 Alacant/Alicante, Spain; joseluis.pastor@ua.es (J.L.P.); fjis@alu.ua.es (F.J.I.); isidro.sanchez@ua.es (I.S.); ma.climent@ua.es (M.Á.C.); 2 Departamento de Ingeniería Civil, Urbanismo y Aeroespacial, Escuela de Arquitectura, Ingeniería y Diseño, Universidad Europea, c/Tajo s/n, 28670 Villaviciosa de Odón, Madrid, Spain; mariadolores.esteban@universidadeuropea.es (M.D.E.); raulruben.rodriguez@universidadeuropea.es (R.R.R.)

**Keywords:** micropiles, sustainability, special geotechnical works, impedance spectroscopy, microstructure, compressive strength, fly ash, ground granulated blast furnace slag, sulphate attack, cement grouts

## Abstract

Nowadays, one of the most popular ways to get a more sustainable cement industry is using additions as cement replacement. However, there are many civil engineering applications in which the use of sustainable cements is not extended yet, such as special foundations, and particularly micropiles, even though the standards do not restrict the cement type to use. These elements are frequently exposed to the sulphates present in soils. The purpose of this research is to study the effects in the very long-term (until 600 days) of sulphate attack in the microstructure of micropiles grouts, prepared with ordinary Portland cement, fly ash and slag commercial cements, continuing a previous work, in which these effects were studied in the short-term. The microstructure changes have been analysed with the non-destructive impedance spectroscopy technique, mercury intrusion porosimetry and the “Wenner” resistivity test. The mass variation and the compressive strength have also been studied. The impedance spectroscopy has been the most sensitive technique for following the sulphate attack process. Considering the results obtained, micropiles grouts with slag and fly ash, exposed to an aggressive medium with high content of sulphates, have shown good behaviour in the very long-term (600 days) compared to grouts made with OPC.

## 1. Introduction

Nowadays, the sustainability has a major importance in the cement industry, and the main aim in that regard is to reduce the CO_2_ emissions produced during cement manufacturing. Among the several ways to lessen these emissions, the use of additions as cement replacement has become very popular in recent years [1,2,3,4,5]. In general, many of these additions are wastes coming from other industries, so their reuse also has an added benefit. Furthermore, some of them can react directly with water or with portlandite formed during the cement hydration, producing new hydrated products which improve the properties of cement-based materials [6,7,8]. They are called active additions, and fly ash and ground granulated blast furnace slag are two of the most popular ones. Many studies show that cementitious materials with ground granulated blast furnace slag and fly ash develop a denser pore structure of concrete at later ages [4,8,9] and consequently they show very good durability properties in the long-term [8,10], such as their permeability [11] and their resistance to aggressive ion ingress [12,13,14,15].

Therefore, both additions have good behaviour for several applications [8], mainly for marine structures [2,16,17]. Nevertheless, there are many civil engineering fields in which the use of cements with fly ash or ground granulated blast furnace slag is not extended yet, such as the special geotechnical works, with the exception of soil mixing [18,19]. In geotechnical engineering, cements with class C fly ash and alkali activated slag have been frequently used as a binder additive to improve the strength of soils or used as grout or deep soil mixing. Cementitious products CSH/CASH fill voids and act as chemical bonds that connect soil particles and grouts together, thus the strength increases at macroscale [18,19,20].

During the last years, special geotechnical works have undergone a great development, especially the micropiles. A micropile is a small-diameter (less than 300 mm) cylinder-shape foundation drilled into the soil usually grouted with cement grout, although mortars are also sometimes used, and reinforced with steel tubes or ribbed bars [21,22,23]. Micropiles are commonly used to transfer loads from structures to deep strata when shallow soil is too soft to support those loads. In relation to the cement type used for preparing the micropiles grouts, the micropiles standards [21,22,23] do not specify any restriction, provided that the grout reaches a certain compressive strength. Notwithstanding, the cement grouts for micropiles are habitually made using an ordinary Portland cement (OPC), particularly in Spain, even though this application could be a potential field for extending the use of sustainable cements which incorporate fly ash and ground granulated blast furnace slag.

Special geotechnical works, and especially the micropiles, are usually in contact with soils and groundwater, therefore regarding their durability the sulphate attack is one of the most aggressive that this type of structural elements can be exposed to in their service life. This attack is complex [20], entailing first a progressive portlandite dissolution and a CSH phases decomposition [20,24]. Next, expansive ettringite crystals are formed, which fill the pores, causing volumetric strains in the microstructure of the material [25] and producing microcracking. The progressive development of this phenomenon implies a fall of mechanical strength of the materials and a loss of durability [26]. The chemical reactions produced by the sulphate attack are shown in Equations (1) and (2).

Ca(OH)_2_ + Na_2_SO_4_ + 2H_2_O → CaSO_4_·2H_2_O + 2NaOH (1)

3CaO·Al_2_O_3_ + 3CaSO_4_·2H_2_O + 26H_2_O → 3CaO·Al_2_O_3_·3CaSO_4_·32H_2_O (2)

In recent research works [27,28,29,30], it has been observed a good performance in the short-term of sustainable grouts for micropiles prepared with cements which incorporate fly ash and ground granulated blast furnace slag, hardened immersed in distilled water and also exposed to a sulphate solution, in relation to their microstructure and compressive strength. It is well-known that the microstructure of cement-based materials is directly related to their service properties [31,32]. In one of the abovementioned researches [29], the changes in the microstructure of the grouts due to the exposure to sulphate solution were successfully characterised until 120 exposure days using the novel non-destructive impedance spectroscopy technique [33,34,35], being the first experience in which this technique was used for detecting the effects of sulphate attack when cements which incorporate fly ash and slag are used.

Despite this good behaviour at relative early times of exposure to sulphate attack, it is very interesting to study the evolution in the long-term of the microstructure and properties of slag and fly ash cement grouts in contact with this aggressive ion, which could be a more realistic datum to be applied to the service life of real micropiles. In addition, the fact that the reinforcement bars and pipes of this kind of special geotechnical work are embedded in cement grouts, instead of concrete, could involve a different performance of these elements against the attack of aggressive substances, mainly if cements with additions are used.

Therefore, the present research continues that previously mentioned work [29], but now the main objective is to study the effects in the very long-term (until 600 days) of sulphate attack in the microstructure of grouts for micropiles, prepared with fly ash and slag commercial cements, compared to OPC ones. The microstructure changes have been analysed with the non-destructive impedance spectroscopy technique, whose results were compared with those obtained using the classical destructive mercury intrusion porosimetry technique and the well-known non-destructive “Wenner” resistivity test. Furthermore, the mass variation and the evolution of compressive strength of the grouts during the 600 days period have also been studied, due to their importance in relation to the requirements of micropiles standards [21,22,23]. Moreover, both are common parameters used in the literature for checking the behaviour of cementitious materials kept in sulphate medium [20,36,37,38]. Finally, the analysis of compressive strength is also interesting because the micropiles mainly transfer axial loads to bearing stratum.

## 2. Materials and Methods

### 2.1. Sample Preparation

In this research, cement grouts were analysed. They were prepared using three different commercial cements to approach real in situ conditions of micropiles construction, in which the blend of ordinary Portland cement and additions would be very difficult to prepare for grouting these elements. In the first place, an ordinary Portland cement, CEM I 42.5 R [39] (CEM I hereafter), was used. Furthermore, grouts prepared with two sustainable cements, which incorporate active additions, were studied. On the one hand, a ground granulated blast-furnace slag cement, a type III/B 42.5 L/SR [39] (labelled CEM III hereafter) was used, whose content of slag was between 66% and 80% of total binder. On the other hand, grouts were also made with a fly ash cement, type CEM IV/B(V) 32.5 N [39] (CEM IV from now on), with a content of this addition ranging from 36% to 55% of total binder. The different components of each one of commercial cements and its percentage of the total binder are detailed in Table 1. The water to cement ratio was 0.5 for all the grouts, achieving the requirements of micropiles standards [21,22,23].

Two types of cylindrical specimens were prepared, which were cast in moulds of 10 cm diameter and 15 cm height and in moulds of 7.5 cm diameter and 30 cm height, respectively. Moreover, prismatic samples with dimensions 4 cm × 4 cm × 16 cm were also made [40]. All the specimens were cured for 7 days in a temperature and humidity controlled chamber at 20 °C and 95% RH. Once finished this period, they were de-moulded and the 15 cm-height cylindrical samples were cut to obtain slices of approximately 2 cm thickness. Besides, the 4 cm × 4 cm × 16 cm samples were also cut in three prisms of dimensions 4 cm × 4 cm × 5.3 cm approximately. After that, all the samples were exposed to the aggressive medium.

### 2.2. Exposure Medium

When the curing period had finished, all the specimens were exposed to an aggressive sulphate medium, which consisted of 15% by weight of reagent grade anhydrous sodium sulphate (Na_2_SO_4_) aqueous solution. The exposure period was until 600 days to study the behaviour of the grouts regarding the attack of this aggressive substance in the long-term. The aqueous sodium sulphate solution was changed every 60 days during the test period. The volume of sulphate solution was approximately 4 times the volume of the samples, as recommends the ASTM C 1012-04 standard [41].

### 2.3. Impedance Spectroscopy

The non-destructive impedance spectroscopy technique has many advantages compared to other classical techniques, because it allows registering the microstructure changes experienced by the same sample during a time interval, as well as getting global data of its pore network. Recent studies have employed this technique for following the development of the pore structure cement-based materials, although in the majority of them ordinary Portland cement was used [4,33,42,43]. Regarding the study of the microstructure evolution of cement-based materials hardened in contact with a sulphate medium using impedance spectroscopy, the only experience is the author’s previous recent work [29] in which OPC, fly ash and slag cement grouts were studied until relatively low exposure times (120 days). Then, there is no experience about using this technique for studying OPC, slag and fly ash cement samples exposed to sulphate attack in the very long-term.

**Figure 1:**
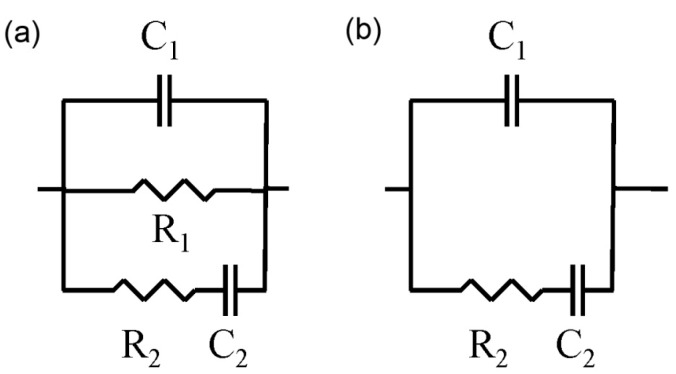
(**a**) Equivalent circuit used for the fitting of the impedance spectra obtained using the contacting method [42]; (**b**) Equivalent circuit used for the fitting of the impedance spectra obtained using the non-contacting method [42].

The impedance measurements were performed using an Agilent 4294A analyzer (Agilent Technologies, Kobe, Japan), which permits capacitance measurements in the range from 10^−14^ F to 0.1 F, with a maximum resolution of 10^−15^ F. The measurements were taken over a frequency range of 100 Hz to 100 MHz. The electrodes used were circular (Ø = 8 cm) and made of flexible graphite, attached to a copper piece with the same diameter. Both contacting and non-contacting methods were used [4,29,42]. The measured data were fitted to the equivalent circuits proposed by Cabeza et al. [42] (see Figure 1), which include two time constants.

In the authors’ previous recent work [29], the validity of these equivalent circuits for OPC, slag and fly ash cement grouts was already checked using the Kramers–Kronig (K-K) relations [44] and the differential impedance analysis [42,45]. As can be observed in Figure 1, the impedance parameters R_2_, C_1_ and C_2_ can be obtained using both contacting and non-contacting methods. However, due to the higher accuracy of non-contacting method, only the R_2_, C_1_ and C_2_ results determined using this method were analysed.

Five different slices of approximately 2 cm thickness were tested for each cement type. The evolution of impedance parameters with time has been reported over a 600-day exposure period, except for CEM I grouts, which could only be measured until 380 days approximately, because at that age these samples were completely destroyed by sulphate attack, preventing further measurements.

### 2.4. Electrical Resistivity

The electrical resistivity gives information about pore connectivity in a cement-based material [46,47]. Here, the Wenner four-point test was used to obtain the resistivity of the grouts, according to the Spanish standard UNE 83988-2 [48]. This parameter was measured using a Proceq analyser on 30 cm-height with 7.5 cm-diameter cylinders until 600 days of exposure to sulphate medium.

### 2.5. Mercury Intrusion Porosimetry

The microstructure of the mortars was also studied using mercury intrusion porosimetry, for checking the non-destructive techniques results. The tests were performed with a Micromeritics Autopore IV 9500 porosimeter (Norcross, GA, USA). Before the test, samples were oven dried for 48 h at 50 °C. The samples were obtained from slices of 2 cm-height. For each age, two measurements were performed on each grout type. Total porosity, pore size distribution and percentage of Hg retained at the end of the experiment were studied. The testing ages were 28, 60, 90, 120, 180, 365 and 600 days.

### 2.6. Mass Variation

The mass variation is a common parameter used in the literature for checking the performance of cement-based materials exposed to aggressive media, such as sulphate attack [49,50]. In this work, the mass variation has been measured until 600 days of exposure to sodium sulphate solution in prisms of dimensions 4 cm × 4 cm × 5.3 cm, which were also used to determine the compressive strength. In particular, the percentage of mass variation with respect to the initial mass of the samples, measured before exposing them to the sodium sulphate solution after the 7-day curing period, was studied.

### 2.7. Compressive Strength

According to the micropiles standards [21,22,23], the grouts have to reach a certain compressive strength, so it is important to study how the sulphate attack affects the evolution of this parameter in the long-term. The compressive strength is also frequently used for studying the damage by sulphates produced in cementitious materials [20,51,52]. Here, the compressive strength was determined in prisms of dimensions 4 cm × 4 cm × 5.3 cm, according to the Spanish standard UNE-EN 196-1 [40]. Three specimens were tested for each cement type. The testing ages were 28, 60, 90, 120, 180, 365 and 600 days. Additionally, for CEM I grouts, the compressive strength was also determined at 500 days, due to the high degree of damage shown by this samples in the very long-term.

## 3. Results

### 3.1. Impedance Spectroscopy

**Figure 2:**
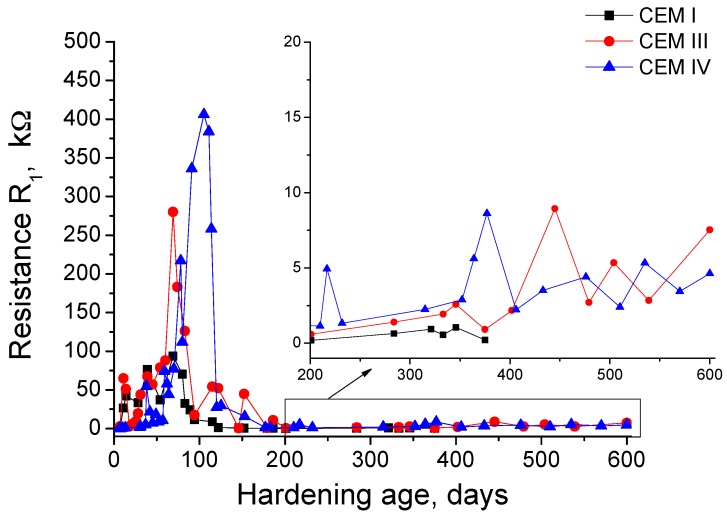
Results of impedance spectroscopy resistance R_1_ for CEM I, III and IV grouts.

The results of resistance R_1_ are shown in Figure 2. In the short-term, this parameter increased for the studied grouts. However, the maximum resistance R_1_ reached value is different depending on the cement type used. The highest R_1_ was observed for CEM IV grouts at 100 hardening days approximately, followed by CEM III ones, whose greatest value was noted at 70 days. Finally, the lowest maximum R_1_ value corresponded to CEM I grouts, and it was also observed at 70 exposure days approximately. Since then, this parameter showed an important fall for all the grouts. On the one hand, for CEM I and III samples, the greatest R_1_ decrease was produced between 70 and 100 days. On the other hand, for CEM IV grouts, this highest fall was observed from 100 to 130 days. Until about 200 days, the R_1_ of CEM III and IV samples continued reducing, although at a lower rate compared to previous ages, whereas it kept practically constant for CEM I grouts. Despite that, the R_1_ values of grouts that incorporate active additions were higher than those observed for CEM I ones. Between 200 and 600 days, in general, an increasing tendency of R_1_ resistance for CEM III and IV has been observed, although during that period the R_1_ values showed several small rises and falls. Finally, as has been already explained, the R_1_ results for CEM I grouts finished at 380 days, when the slices of this type of grout were completely broken due to the effect of sulphate attack, which made it impossible to continue measuring at further ages.

**Figure 3:**
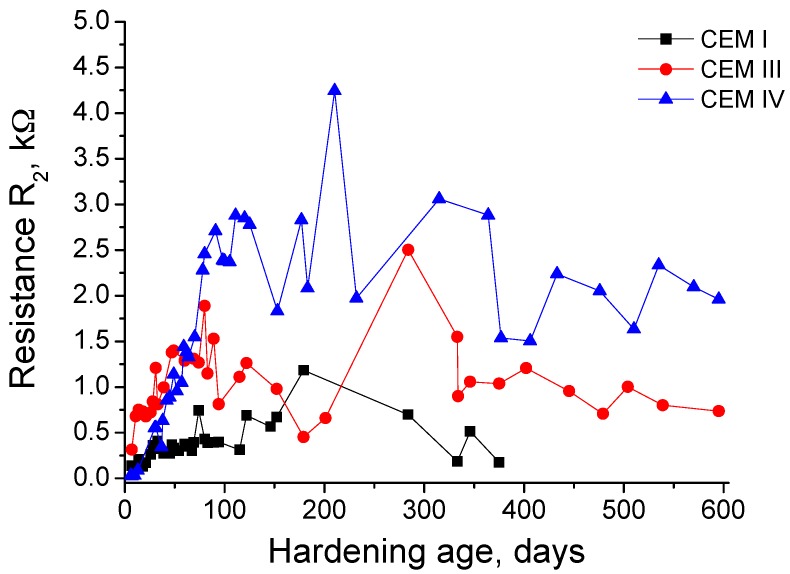
Results of resistance R_2_ for CEM I, III and IV grouts.

The evolution with time of resistance R_2_ is depicted in Figure 3. At initial ages, this parameter was higher for CEM III grouts, followed by CEM I and IV ones. The resistance R_2_ rose relatively fast in the short-term for the three kinds of grouts studied, although the increasing rate was different for each type, as happened with the resistance R_1_ results previously described. The grouts prepared using cements with active additions showed a higher increase of R_2_ than CEM I ones. Since 100 hardening days, in general the increase of resistance R_2_ was slowed down for all the grouts and the greatest values of this parameter were observed for CEM IV ones. For this type of grout, from 100 to 150 days the R_2_ decreased, and from then until 200 days it rose again, reaching at that age the highest R_2_ value for CEM IV samples. Between 200 and 600 days, the resistance R_2_ for this fly ash grouts showed a decreasing tendency, although several increases and falls of this parameter during that period have been noted. On the other hand, for CEM III grouts, the resistance R_2_ decreased from 100 to 200 days, increased from then until 300 days, when it reached its highest value for this cement type, and it fell again at 350 days, keeping practically constant at greater exposure ages. Finally, the CEM I grouts showed a progressive rise of R_2_ until 200 days, falling from then until the breaking of this type of samples. The lowest values of this parameter corresponded to CEM I grouts in the majority of studied period.

**Figure 4:**
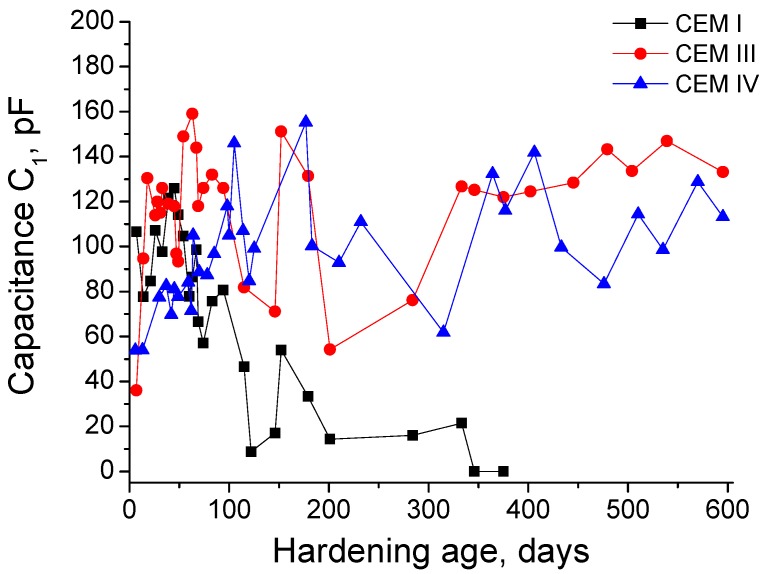
Impedance capacitance C_1_ results for CEM I, CEM III and CEM IV grouts.

Now, the capacitance C_1_ results will be described, which can be observed in Figure 4. At very early ages, the lowest values of this parameter have been observed for CEM IV grouts. In general, the C_1_ increasing rate in the short-term was similar for CEM I and III samples, whereas it was slower for CEM IV ones. For CEM I grouts, the capacitance C_1_ showed a decreasing tendency from 50 days, although it has been observed little rises at 100 and 150 days. For CEM III grouts, this parameter showed the first maximum at 70 days, and from then, C_1_ decreased and after that it rose again, reaching another maximum at 150 days approximately, followed by an important fall at about 200 days, increasing again from then to the end of research. As has already been explained, the capacitance C_1_ for CEM IV grouts showed a slower rise compared to CEM III ones. However, from 120 days, the C_1_ values and their evolution were very similar for both fly ash and slag cement grouts.

**Figure 5:**
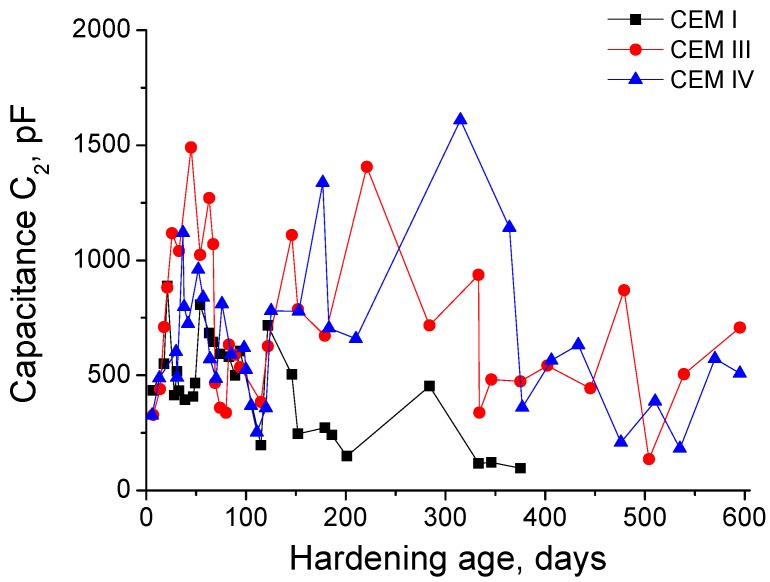
Results of capacitance C_2_ for CEM I, CEM III and CEM IV grouts.

The next impedance parameter results to describe are the capacitance C_2_ ones, which are depicted in Figure 5. This parameter was very similar for all the grouts in the very short-term. As happened with the capacitance C_1_, the C_2_ also increased quicker for CEM III grouts than for CEM IV ones. Moreover, before 50 days, C_2_ values were higher for both types of grout with active additions than for those prepared with CEM I. For CEM III grouts, the capacitance C_2_ reached its first maximum at 50 days, and it fell from that age to 120 days, rising from then until 150 days approximately, when it showed a decreasing trend in general terms. The capacitance C_2_ tendencies observed for CEM IV grouts were very similar to CEM III ones, although the increase of this parameter was slower, and as a consequence, the C_2_ values are lower at relatively early ages. The last C_2_ maximum for CEM IV grouts was observed at about 300 days, and this parameter decreased from then, although from 400 days the values obtained were very similar to those observed for CEM III ones. On the other hand, the capacitance C_2_ of CEM I grouts showed several increases and falls in the studied period, with lower values in the middle-term and in the long-term, compared to those obtained for CEM III and IV ones.

### 3.2. Electrical Resistivity

**Figure 6:**
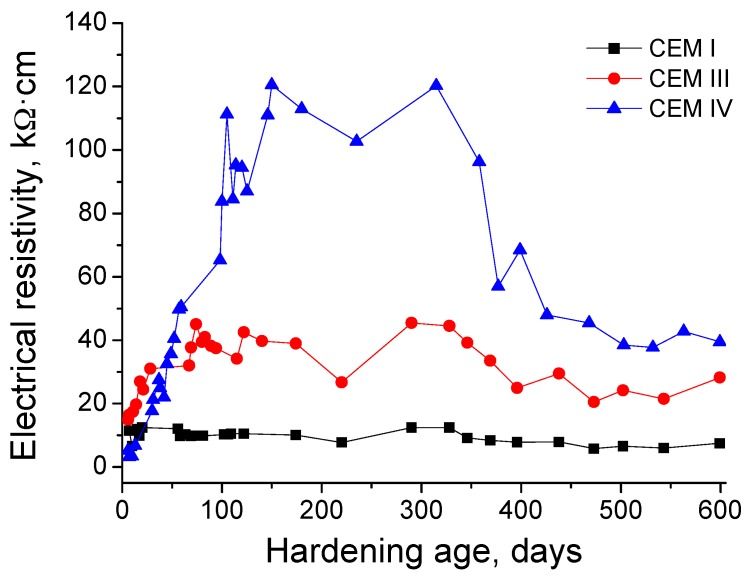
Evolution of electrical resistivity for CEM I, III and IV grouts.

The results of electrical resistivity obtained for the three types of grouts studied are depicted in Figure 6. The lowest resistivity values at very early ages were observed for CEM IV grouts, although they showed a fast increasing rate with time. Until 50 days, the highest resistivity values corresponded to CEM III samples, and from that age, the CEM IV ones showed the greatest resistivity, which reached very high values compared to the rest of studied cement types. The resistivity for CEM IV grouts finished to rise at 200 days, keeping practically constant until 350 days and falling from then, although its values continued being considerably higher than for the rest of studied grouts at 600 days. The evolution of CEM III samples resistivity showed similar tendencies than previously described for CEM IV ones, but with lower values. Finally, the electrical resistivity for CEM I grouts showed small changes with time, and, in general, its values were the lowest of all studied cements.

### 3.3. Mercury Intrusion Porosimetry

**Figure 7:**
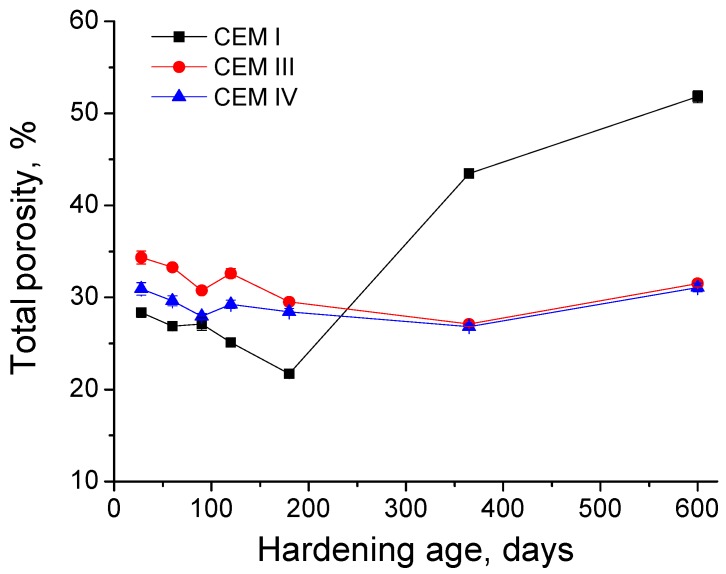
Total porosity results for CEM I, CEM III and CEM IV grouts.

The changes with time of total porosity for the three types of grouts can be observed in Figure 7. From 28 to 180 days, this parameter decreased for all the samples studied. The lowest total porosities in that period were observed for CEM I grouts and the highest for CEM III ones, although the difference between porosity values for all the grout types was not too large. Between 180 and 600 days, total porosity showed very similar values and kept practically constant for grouts with slag and fly ash. On the other hand, in the abovementioned period, this parameter rose highly for CEM I grouts, showing a very great value at 600 days compared to the other kinds of grouts.

**Figure 8:**
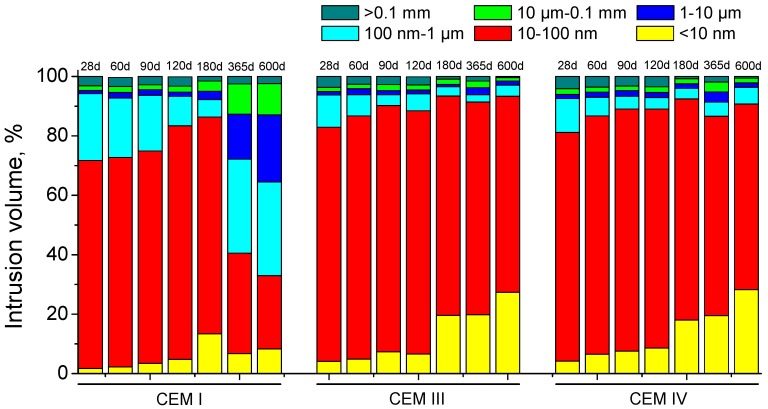
Pore size distributions (in percentage) for CEM I, III and IV grouts.

The pore size distributions obtained for CEM I, III and IV grouts exposed to the aggressive medium are depicted in Figure 8. In general, the main pore size range for the three grouts studied is that comprised between 10 and 100 nm. The most refined microstructure corresponded to CEM III and IV grouts. From 28 to 180 days, a progressive pore refinement has been observed for all the grouts. Since then, the microstructure became a little less refined for slag and fly ash samples, as indicated the small reduction of percentage of pores volume with a size less than 100 nm, but at 600 days the pore network of this CEM III and IV grouts was much more refined than that observed for all previous ages. However, from 180 days, the pore structure of CEM I samples increasingly showed an important loss of refinement, as indicated the important fall of volume of pores with diameters lower than 100 nm.

**Figure 9:**
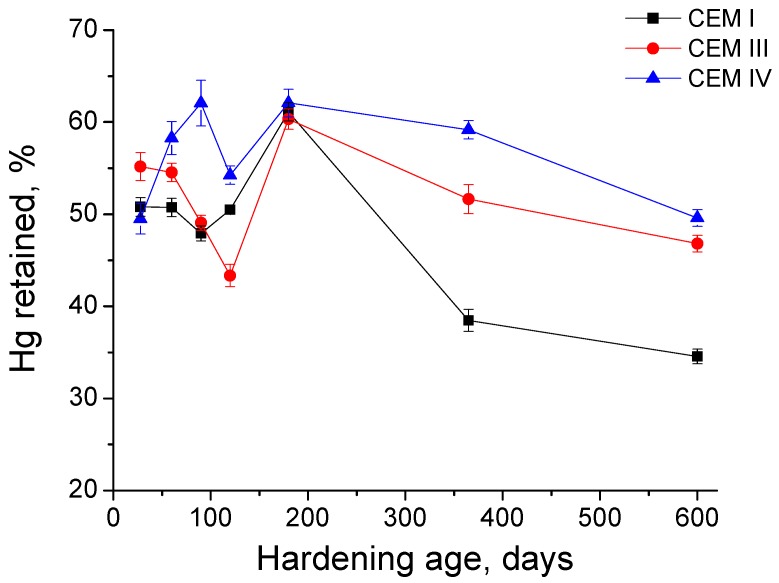
Percentage of Hg retained at the end of experiment for studied grouts.

Regarding the percentage of Hg retained in the samples at the end of the experiment (see Figure 9), at 180 days the value of this parameter was very similar for all the studied grouts. However, until that age, the evolution of this parameter was different depending on the cement type used. First, the Hg retained kept practically constant for CEM I grouts between 28 and 120 days and increased from then to 180 days. For CEM III samples, this parameter decreased until 120 days, and rose at 180 days. Finally, the Hg retained of CEM IV grouts in general grew between 28 and 120 days, with the exception of a little fall observed at 120 days. In the long-term (exposure ages longer than 180 days), this parameter decreased for all the samples, being this reduction more noticeable for CEM I grouts, which showed the lowest Hg retained value at 600 days. This drop was slower for grouts with slag and fly ash, especially for CEM IV ones, although at 600 days the Hg retained was very similar for both CEM III and IV grouts.

### 3.4. Mass Variation

**Figure 10:**
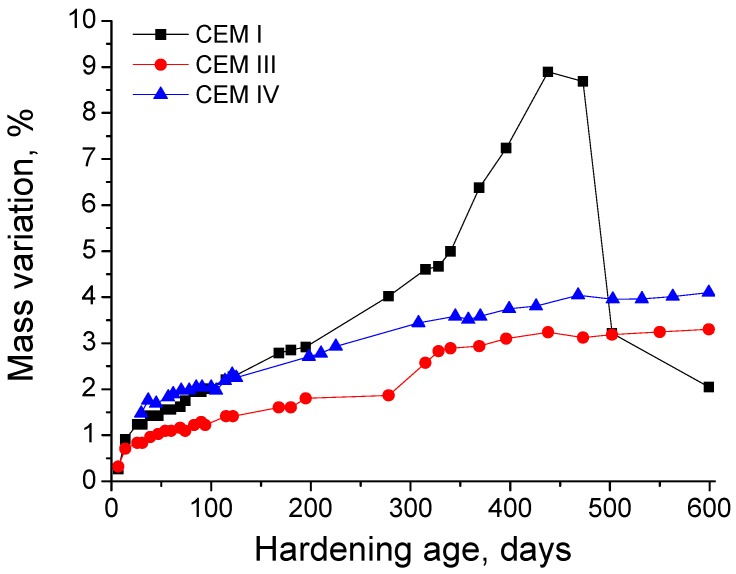
Mass variation (in percentage) observed for CEM I, III and IV grouts in contact with the aggressive medium.

With respect to the mass variation experienced by the grouts due to the contact with the sulphate medium, the changes with time of this parameter can be observed in Figure 10. During the first 300 days, this parameter showed a progressive increase for all the grouts. Nevertheless, from that age, it started to rise quickly for CEM I samples, whereas it tended to stabilize for CEM III and IV ones. The maximum value of mass variation percentage for CEM I specimens was reached at 400 days approximately, when it started to decrease very fast. This great fall in the long-term was not observed for grouts with slag and fly ash.

### 3.5. Compressive Strength

**Figure 11:**
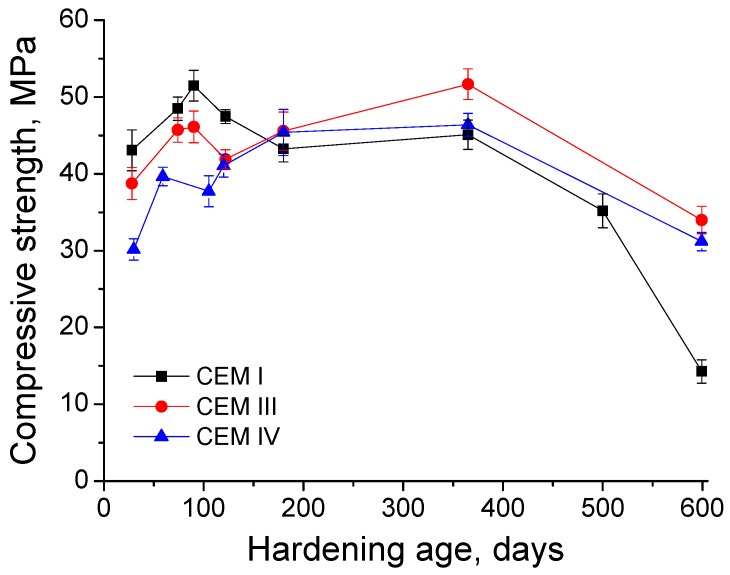
Results of compressive strength obtained for CEM I, CEM III and CEM IV grouts.

**Figure 12:**
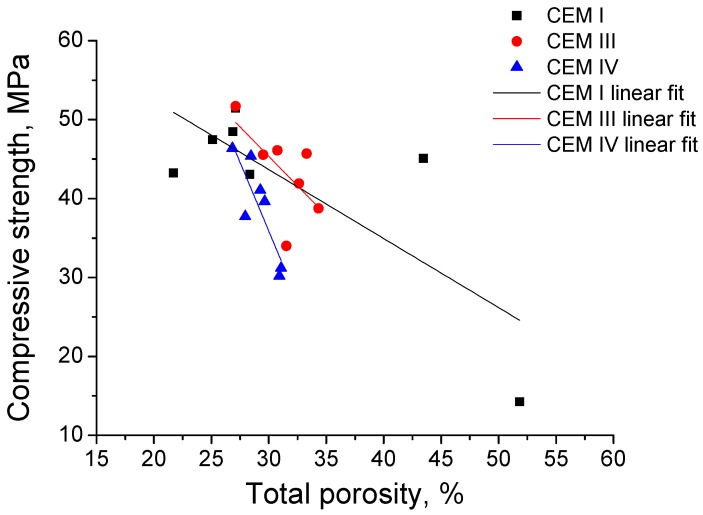
Relationship between compressive strength and total porosity for CEM I, III and IV grouts.

The results of grouts compressive strength are depicted in Figure 11. Until 120 days, the highest compressive strengths were observed for CEM I grouts. For this type of grout, this parameter increased from 28 to 90 days, when it started to decrease, although its major fall has been observed between 365 and 600 days. In general terms, the compressive strength for CEM III grouts rose up to 365 days, except a little drop at 120 days, and fell between 365 and 600 days. The lowest compressive strength values in the short-term were noted for CEM IV grouts, however they developed a progressive increase until 180 days, keeping practically constant at 365 days, and decreasing from then to 600 days. The compressive strengths in the very long-term (600 days) were very similar for slag and fly ash cement grouts and higher than those observed for CEM I ones. Finally, the relationship between compressive strength and total porosity is represented in Figure 12. As can be observed in this figure, the compressive strength decreased when the total porosity increased.

## 4. Discussion

Regarding the impedance spectroscopy results, the resistances R_1_ and R_2_ are related to the electrolyte which fills the pores of the sample [42], so it could be expected that both parameters change when the pore structure of the grouts evolves due to development of hydration and pozzolanic reactions or as a consequence of the effects produced by the sulphate attack.

On the one hand, the resistance R_1_ is associated only with the percolating pores of the sample [42]. The increasing tendency of resistance R_1_ (see Figure 2) observed at early ages for all cement types would indicate a gradual microstructure refinement, which could be due to the development of cement and slag hydration and fly ash pozzolanic reactions. At those early ages, the sulphate attack has hardly started, so its effects in the microstructure are not observed yet. The maximum values of resistance R_1_ were higher for CEM III and IV grouts than for CEM I ones, which would indicate that the microstructure would be more refined for grouts with active additions [4] compared to those prepared with ordinary Portland cement.

The resistance R_1_ grew faster for CEM III samples than for CEM IV ones. This result could be explained in relation to the different performance of slag and fly ash. The slag reacts directly with the water since the mixing of the grouts [8,31], so the slag hydration initiates immediately and then, its benefits in the pore network of the grouts are produced sooner. However, the pozzolanic reactions of fly ash start once enough portlandite has been formed as a product of cement hydration [6,7], so these pozzolanic reactions are delayed compared to slag and cement hydration, and therefore it is necessary more time to observe the effects of fly ash in grouts microstructure. For that reason, the maximum R_1_ for CEM IV samples was observed at a later age than for CEM III samples.

At different ages in the period between 70 and 100 days, depending on the cement type, the resistance R_1_ of the grouts started to fall. The decrease of this parameter would show a loss of solid fraction and pore refinement, probably as a consequence of the appearance of expansive products produced during sulphate attack [20,24,26,53], which would crack the current porous network. The fact that the effects of sulphate attack in resistance R_1_ have been observed later for CEM IV grouts than for the rest, could also be related to the abovementioned delay of fly ash pozzolanic reactions, because the formation of solid phases as products of those reactions could counteract the loss of solid fraction at initial stages of sulphate attack. Finally, the small rises and decreases of resistance R_1_ observed for slag and fly ash grouts in the very long-term (from 300 days) could indicate the continuous formation of expansive products, which first would occupy the pre-existing pores and spaces produced by the previous cracks, refining the network and increasing the resistance R_1_, until they have filled all the available spaces in the microstructure, when they would break it again, entailing a decrease of resistance R_1_. The R_1_ results would suggest that this process could have happened repeatedly at very high ages.

On the other hand, the resistance R_2_ is related to all the pores of the sample [42]. In general, its results (see Figure 3) are in keeping with those obtained for resistance R_1_, such as the greatest R_2_ values observed for fly ash and slag cement grouts compared to CEM I ones, which would reveal once again the higher pore network refinement produced by these active additions. Nevertheless, there are some differences between the results of resistances R_1_ and R_2_. The maximum R_2_ values were reached later than the maximums resistances R_1_ for the three types of studied grouts. Moreover, the fall of resistance R_2_ at greater ages was not as pronounced as the decrease noted for resistance R_1_. This also happened for all the grouts, so it suggests that sulphate attack has less effect in resistance R_2_ than in R_1_.

This different behaviour of both resistances could be related to the fact that the resistance R_1_ gives information about the percolating pores of the sample [42], and the resistance R_2_ is associated with all the pores of the sample [42], that is, both percolating and occluded ones. The percolating pores are directly accessible by the sodium sulphate solution, so it is expected that the sulphate attack is produced earlier and quicker in them, and it would also be more severe. Therefore, while in these percolating pores the hydration and pozzolanic reactions would still be developing, the first stages of sulphate attack would be starting. On the contrary, the occluded pores would not receive directly the sulphate attack, so no damages of this attack would be produced in them or it would happen at later ages, when the microcracking phenomenon caused by expansive products in percolating pores has created new spaces in the microstructure, which would make accessible part of the initial occluded porosity. As a consequence, in this occluded pores the main phenomenon produced would be the new solids formation due to the development of hydration and pozzolanic reactions, which would contribute to increase the resistance R_2_, counteracting the fall of this parameter produced by the damages in the percolating pores caused by the sulphate attack, at least until middle-term ages. This could explain the delay in detecting the effects of this attack between the impedance resistances R_2_ and R_1_. Finally, as has been noted for resistance R_1_, the R_2_ values also showed several rises and decreases in the very long-term (from 300 days), which could point out the alternation of filling and cracking processes in the pores, linked to the formation of sulphate expansive products.

Now, the results of impedance capacitance C_1_ will be discussed (see Figure 4). This capacitance is associated to the solid fraction in the specimen [42]. The initial growth of this parameter in the studied grouts is in agreement with previously discussed impedance resistances results, and it could be related to the formation of solid phases due to the cement and slag hydration and fly ash pozzolanic reactions [4,27,28]. In addition to this, the slower increase of capacitance C_1_ for CEM IV grouts also coincides with R_1_ and R_2_ results, revealing the abovementioned delay of fly ash pozzolanic reactions in comparison with slag and cement hydration [7]. Until 100 days, the capacitance C_1_ showed a similar magnitude for all the grouts studied, which would suggest that they would have a similar solid fraction, independently of the refinement degree of their pore network, and consequently only little differences in porosity between them would be expected. The decrease of capacitance C_1_ produced in the middle-term would indicate a loss of solid fraction, which could be related to the effects of sulphate attack, as has been explained for impedance resistances results. This C_1_ fall was more important for CEM I grouts, and this would mean that the damages by sulphates would be much more severe for this cement type samples than for slag and fly ash ones.

The impedance capacitance C_2_ is related to the pore surface in contact with electrolyte which fills the pore network of the material [54]. In general, the results obtained for this parameter (see Figure 5) showed coincidences with those observed for capacitance C_1_ and resistances R_1_ and R_2_, already described and discussed. The rise of capacitance C_2_ at early ages would mean an increase of the pore surface, probably due to the formation of CSH gel layers [54] as products of hydration and pozzolanic reactions. This result would indicate a progressive pore refinement, which would be more important in CEM III and IV grouts, as suggests their higher C_2_ values in the short-term compared to those noted for CEM I ones, being in accordance with the rest of impedance spectroscopy parameters.

In relation to the comparison between capacitances C_1_ (see Figure 4) and C_2_ (see Figure 5), it was noted that generally the C_2_ fell earlier than the C_1_ in the exposure period previous to 100 days. This fact was well-observed especially for CEM III and IV grouts and it could be related to the formation of sulphate attack products. On the one hand, these products would progressively break the rough structures deposited on the pore surface and produced by the hydration and pozzolanic reactions, entailing a decrease of the specific surface of the pores and the subsequent fall of capacitance C_2_. Furthermore, when the silting of the pores produced by the formation of ettringite is more advanced, the pores would be getting increasingly filled, so the pore surface could be reduced, which also contribute to the reduction of capacitance C_2_. On the other hand, both abovementioned processes would produce an increase of total solid fraction of the samples, which would also raise the capacitance C_1_. For these reasons, there are periods of time in which the capacitance C_1_ was still increasing, while the capacitance C_2_ was decreasing.

Nevertheless, once the pores were completely filled of ettringite and other expansive products formed during the sulphate attack, a microcracking of the microstructure and a loss of solid fraction in the material would be produced. Particularly, the loss of solid fraction would entail a fall of capacitance C_1_ values and the formation of microcracks would increase the capacitance C_2_, because these microcracks would also constitute new pore surface. Once again, the comparison of both capacitances C_1_ and C_2_ results would give information about that hypothesis. Specifically, the evolution of capacitance C_1_ (see Figure 4) showed noticeable minimums at about 100 days for CEM I grouts, at 200 days for CEM III grouts and at 300 days for CEM IV grouts, which would mean a relatively important cracking and loss of material. Consequently, an increase of specific surface in the pore structure due to this microcracks would be expected, which would involve a fast rise of capacitance C_2_. Then, if the C_2_ results are consulted (see Figure 5), it can be observed a sudden increase of this parameter at about 100 days for CEM I grouts, at 200 days for CEM III grouts and at 300 days for CEM IV grouts, which completely coincides with the C_1_ falls and would give support to the hypothesis of the development of abovementioned processes in the microstructure of the samples. Therefore, it is important to emphasize that this combined analysis of impedance capacitances C_1_ and C_2_ permits to obtain information about microcracking processes by sulphate attack in the pore network of cement-based materials.

The electrical resistivity results (see Figure 6) showed coincidences with those obtained for impedance spectroscopy parameters, especially with resistance R_2_ ones. At initial ages, the lowest resistivity values were observed for CEM IV grouts, which would indicate again the delay of fly ash pozzolanic reactions compared to slag and cement hydration, previously explained. In spite of that, generally the electrical resistivity was higher for slag and fly ash grouts than CEM I ones. This result is in agreement with impedance spectroscopy ones, and it would confirm that the pore network is more refined when cements with active additions are used for preparing the grouts. However, in relation to sulphate attack, more time was needed to detect its effects using the electrical resistivity (ages higher than 300 days), and they were not as clear as has been observed with impedance spectroscopy.

In a previous work [29], where the performance of sustainable cement grouts was studied until relatively early ages, and which is continued by this new research, it was pointed out that practically no effects of sulphate attack were observed using Wenner four-point electrical resistivity test, compared to impedance spectroscopy technique. On the one hand, this result was related to the different geometry of samples used for each technique, which entailed the double of relationship surface/volume for impedance spectroscopy samples than for resistivity ones [29]. On the other hand, it was noted that the fact that the Wenner four-point test is a superficial technique, while the impedance spectroscopy is a global technique, which permits measurements through the samples, could have an influence in the differences between the results obtained with both techniques [29]. Therefore, in view of the electrical resistivity measurements until 600 days of exposure to sulphate media, it would be confirmed that the Wenner four-point test is not the most suitable technique for studying the effects in the long-term of sulphate attack in the microstructure of the grouts. On the contrary, the impedance spectroscopy technique seems to allow getting more reliable information of the evolution of microstructure of the grouts while the sulphate attack is developing.

Regarding the mercury intrusion porosimetry, in general the total porosity (see Figure 7) was reduced for all the grouts at early ages, which would be in keeping with already discussed results, and it could be related to the solid phases formation as products of hydration and pozzolanic reactions. In addition to this, until 180 days there were not too many differences between the total porosities observed for the different types of analysed grouts, which would indicate that the solid fraction was similar in all of them, coinciding with the similar magnitude showed by impedance capacitance C_1_. Since 180 days, it has been noted an important rise of total porosity for CEM I grouts, which continued until 600 days. This increase could be produced by a severe sulphate attack. For CEM III and IV grouts, the total porosity kept practically constant from 180 to 600 days, so no effects of sulphate attack are revealed by this parameter.

The progressive pore refinement in the short-term observed in all pore size distributions of the grouts (see Figure 8), and the fact that the volume of smaller pores was higher for CEM III and IV samples, also coincides with impedance spectroscopy and electrical resistivity results. However, as happened for total porosity, the effects of sulphate attack in the pore size distribution have only been noted for CEM I grouts, in which an important loss of microstructure refinement was observed at 365 and 600 days, while the pore network even became more refined for CEM III and IV samples.

In relation to the Hg retained in the sample once the mercury intrusion porosimetry test has finished, it provides information about the tortuosity of the pore structure [42]. The changes of this parameter at early ages (see Figure 9) showed the evolution of the pore network, mainly produced by the development of hydration and pozzolanic reactions. At 180 days, all types of specimens had practically the same Hg retained, and this could be related to the formation of expansive products as a consequence of sulphate attack, which would initially increase the tortuosity of the pore network before filling considerably the pores. Between 180 and 600 days, the Hg retained fell for all the grouts. This decrease was more considerable for CEM I grouts, and it would be due to the cracking of the microstructure produced by the expansive products, which would reduce the tortuosity and also the pore refinement, as suggested the pore size distributions already discussed. For CEM III and IV, the decrease of Hg retained was lower, and it could be mainly related to the progressive silting of the pores by the sulphate attack products formation, which would also reduce the pore network tortuosity, and in lesser extent to the microcracking phenomenon, because the pore size distributions of these grouts did not reveal a noticeable loss of microstructure refinement.

Although at early ages the mercury intrusion porosimetry results were similar to those obtained with the rest of techniques used in this research, in the long-term its results did not correlate well with the other microstructure results observed, especially with the impedance spectroscopy ones, which seems to be the most sensitive technique for detecting the processes of sulphate attack according to the results obtained until now. Several examples of this no coincidence were for example the no rise of porosity and no loss of pore refinement for slag and fly ash grouts at greater ages, and in general the later or scarce detection of evidences related to sulphate attack using MIP, depending on cement type. These results would confirm those obtained in the previous work [29] to this research, in which MIP results were not also as clear as impedance spectroscopy ones. This fact could be related to the general drawbacks of mercury intrusion porosimetry [55,56], such as the fact that all pores greater than 900 µm were not registered by the apparatus, and the intrinsic limitations of this technique, which overestimates the volume of smaller pores present in the samples, according to some authors [56]. However, in view of the results obtained in this work for CEM I grouts, when the sulphate attack is severe and very extended, MIP technique could give some information about the effects of this attack in the microstructure. Despite that, considering the discussion of microstructure characterisation explained up to now, the mercury intrusion porosimetry, which is the most used destructive technique for the pore network characterisation of cementitious materials, seems not to be useful in this case, while the new non-destructive impedance spectroscopy technique permits to study with higher trustworthiness the microstructure changes in the grouts due to sulphate attack.

Finally, despite the differences between the techniques used, from the point of view of the microstructure characterisation, all of them coincided in that the micropiles grouts prepared using slag and fly ash cements and exposed to an aggressive medium with high content of sulphates, have a better behaviour in the very long-term compared to grouts made with ordinary Portland cement.

With respect to the mass variation of the samples produced by the contact with sodium sulphate solution (see Figure 10), the main changes with time were observed for CEM I grouts. Until 200 days approximately, the mass increasing rate of this type of grout did not differ too much to those observed for CEM III and IV grouts, and it could be related to the development of cement hydration simultaneously with an initial formation of ettringite and expansive sulphate products. From 200 and 350 days, the mass increasing rate for CEM I grouts became higher, which could be due to the generalization of sulphate attack with more formation of expansive phases, closing progressively the microstructure and increasing the mass of the samples. Between 350 and 450 days, this CEM I samples mass increasing rate continued rising, so the pores silting would go on. Finally, at 450 days, the mass of CEM I grouts started to decrease, because the pore network could not accommodate more expansive phases, and due to the severity of sulphate attack, the cracking and damages in the material were extended, producing the subsequent loss of material. This previously explained process would be in keeping with other authors [57]. On the other hand, for CEM III and IV grouts, a continuous increase of mass has been observed until 600 days, maybe produced first by the development of slag hydration and fly ash pozzolanic reactions and later by the formation of products of the sulphate attack. Therefore, according to these results for CEM III and IV grouts, although at microstructural scale effects of sulphate attack have been observed, it seems that they did not produce a remarkable damage at macroscopic scale, at least regarding the loss of material.

Lastly, the compressive strength results (see Figure 11) until 90 days are controlled by the strength type of each cement, then the greatest values of this parameter corresponded to CEM I grouts, which is the only high early strength cement used, followed by CEM III grouts (the slag cement was a low early strength type) and CEM IV ones (the fly ash cement was the lowest strength type). In spite of that, from 180 days the compressive strength was higher for slag and fly ash cement grouts than for CEM I ones. It is important to note that the growth of this parameter for CEM IV grouts was slower at early ages, which is in agreement with microstructure characterisation results, and would show the delay of fly ash pozzolanic reactions development. In relation to the sulphate attack effects in the compressive strength, it started to decrease at 90 days for CEM I grouts, although its main fall was produced from 365 to 600 days, when the compressive strength was only approximately a 27% of the maximum value of this parameter, reached for this CEM I grouts at 90 days, so there has been a severe loss of strength for these samples. In contrast, the decrease of compressive strength for CEM III and IV grouts was produced only between 365 and 600 days, and the value of this parameter at that age for both cement types was about the 67% of their highest strengths, observed at 365 days. Then, the grouts prepared with CEM III and IV, hardened in contact with a sulphate medium, showed better compressive strength performance in the very long-term compared to CEM I grouts.

## 5. Conclusions

The main conclusions that can be drawn from the results previously discussed can be summarized as follows:

- The pore network of slag and fly ash cement grouts exposed to sodium sulphate medium was more refined than that observed for CEM I ones during the entire period of time studied (until 600 days).
- At early ages, all the grouts showed a progressive pore refinement, independently of cement type used, which has been related to the development of cement and slag hydration and fly ash pozzolanic reactions, which produce new solid phases. Furthermore, the initial formation of expansive products due to the still incipient sulphate attack could also influence this pore refinement in the short-term.
- The growth of compressive strength and the pore structure refinement of fly ash cement grouts were developed in a slower way compared to slag and ordinary Portland cement ones. This result can be explained as consequence of the delay of fly ash pozzolanic reactions with respect to slag and cement hydration.
- The differences between the results obtained for impedance spectroscopy resistances R_1_ and R_2_ could be related to the different degree of damage produced by sulphate attack in the percolating pores and in the occluded pores of the grouts microstructure.
- The combined analysis of impedance spectroscopy capacitances C_1_ and C_2_ permits obtaining information about microcracking processes produced by sulphate attack in the pore network of cement-based materials.
- According to the results obtained, the non-destructive impedance spectroscopy seems to be the most sensitive technique for detecting the processes developed during the sulphate attack in the microstructure of slag, fly ash and ordinary Portland cement grouts for micropiles in the very long-term (until 600 days). This would confirm the good results obtained in a previous work using this technique for the same types of grouts and condition in the short-term (until 120 days).
- Mercury intrusion porosimetry and Wenner four-point electrical resistivity test appear to have limitations when they are used to monitor changes in the microstructure of cement grouts resulting from sulphate attack, at least when this attack is not very extended.
- Although at microstructural scale effects of sulphate attack in slag and fly ash cement grouts have been observed, it seems that they did not produce a remarkable damage at macroscopic scale in the very long-term (600 days), at least regarding the loss of material. On the contrary, the cracking and damages made by this attack on CEM I grouts are severe, producing an important loss of material at 600 exposure days.
- The sulphate attack entailed a severe loss of compressive strength for CEM I grouts in the very long-term, while those prepared slag and fly ash cements showed better compressive strength performance at 600 days.
- Considering the results obtained in this research, micropiles grouts prepared using slag and fly ash cements and exposed to an aggressive medium with high content of sulphates, have a good behaviour in the very long-term (600 days), compared to grouts made with ordinary Portland cement.

## Figures and Tables

**Table 1:** Components of the commercial cements used.

Component	CEM I		CEM III		CEM IV	
	UNE-EN 197-1 Standard [39]	Manufacturer Data ^1^	UNE-EN 197-1 Standard [39]	Manufacturer Data ^1^	UNE-EN 197-1 Standard [39]	Manufacturer Data ^1^
Cement	95–100%	95%	20–34%	31%	45–64%	50%
Limestone	-	5%	-	-	-	-
Blast-furnace slag	-	-	66–80%	69%	-	-
Fly ash	-	-	-	-	36–55%	50%

**^1^** Specific percentage of each component usually used according to the manufacturer.
